# THE RELATIONSHIP BETWEEN SKIPPING BREAKFAST AND DEMENTIA: A RETROSPECTIVE COHORT STUDY IN OSAKA

**DOI:** 10.1093/geroni/igac059.2261

**Published:** 2022-12-20

**Authors:** Hitomi Chikama, Reiko Kanaya, Yasushi Takeya, Hiroshi Toki, Ryohei Yamamoto, Miyae Yamakawa

**Affiliations:** Osaka University, Suita City, Osaka, Japan; Osaka University, Osaka, Osaka, Japan; Osaka University, Suita, Osaka, Japan; Health and Counseling Center, Suita, Osaka, Japan; Osaka University, Suita, Osaka, Japan; Osaka University, Suita, Osaka, Japan

## Abstract

In Japan, annual medical checkups are carried out to prevent lifestyle diseases. Studies have found that skipping breakfast is a risk factor for diabetes, and potentially also dementia. This retrospective cohort study aimed to evaluate the relationship between skipping breakfast and dementia in people with diabetes. The eligible cohort was anyone on the National Health Insurance Database of Osaka who had a checkup in FY 2013 and had diabetes (N = 283,410). The cohort was divided into two groups, those who skipped breakfast more than three times a week (7.38%) and those who did not. The database does not allow individuals to be identified. People with diabetes were defined as those prescribed diabetes drugs in the year before the checkup or with HbA1c of 6.5% or higher at the checkup. People with dementia were defined as those prescribed anti-dementia drugs between April 2014 and December 2017. Data also included body mass index (BMI), smoking, sex , age, and prescription of hypolipidemic or blood pressure drugs. Logistic regression analysis was used, with odds ratio (OR) and 95％ confidence interval . Being male, younger, smoking, having lower BMI or HbA1c levels, and drug prescriptions were associated with skipping breakfast. Skipping breakfast was associated with dementia (OR 1.26, 1.14–1.41), as was lower BMI and being older. For people with diabetes, skipping breakfast is a risk factor for obesity and dyslipidemia, which are associated with dementia. This study therefore provides evidence for a health behavior approach to eating breakfast in people with diabetes.

